# A Cross-Sectional Study on the Quality of Life of Adults With Sickle Cell Disease Followed-Up in Outpatient Clinics: A Single-Center Experience

**DOI:** 10.7759/cureus.73970

**Published:** 2024-11-19

**Authors:** Omar Alzahrani, Ehab Hanafy, Mona Alatawi, Ali M Alferdos, Osama Mukhtar, Aydah Alhowiti, Samira Alomrani

**Affiliations:** 1 Prince Sultan Oncology Center, King Salman Armed Forces Hospital, Tabuk, SAU; 2 Family Medicine, King Salman Armed Forces Hospital, Tabuk, SAU; 3 Preventive Medicine, King Salman Armed Forces Hospital, Tabuk, SAU; 4 Health Education, King Salman Armed Forces Hospital, Tabuk, SAU

**Keywords:** adult patients, mental health, pain management, physical function, quality of life, sickle cell anemia, social impact

## Abstract

Background

Sickle cell disease (SCD) is a genetic blood disorder characterized by abnormal hemoglobin S, leading to red blood cell deformities, chronic hemolysis, and frequent vaso-occlusive crises (VOC). While advancements in medical care have improved survival rates, adults with SCD continue to face substantial challenges in their quality of life (QoL) due to chronic pain, recurrent VOCs, and various complications. This study aimed to evaluate the health-related quality of life (HRQoL) in adult patients aged 14 years and above with SCD and identify key factors influencing patient outcomes using the Adult Sickle Cell Quality of Life Measurement Information System (ASCQ-Me).

Methods

A cross-sectional study was conducted at the Prince Sultan Oncology Center, King Salman Northwest Armed Forces Hospital, Tabuk, Saudi Arabia, between December 2019 and May 2020. The study population comprised adult SCD patients attending outpatient clinics. The QoL was assessed using the ASCQ-Me Short Form, which evaluates five domains: emotional impact, social functioning, pain impact, sleep impact, and stiffness impact. Additionally, a nine-item SCD Medical History Checklist was used to evaluate complications and treatments. Data were collected through structured one-on-one interviews. Scores were transformed into T-scores (mean = 50, standard deviation (SD) = ±10) based on standardized guidelines. Inferential analysis was conducted to compare QoL domains between groups stratified by VOC frequency and severity using the Mann-Whitney U test, with a significance threshold of p ≤ 0.05.

Results

A total of 53 adult SCD patients were surveyed, with the majority aged 25-34 years (50%). Gender distribution was nearly equal, with 50.9% male participants. The prevalence of complications was notable, with 31% reporting chronic pain, 26% reporting gallstones, and 9% reporting avascular necrosis. Laboratory findings revealed a mean hemoglobin level of 8.63 g/dL (SD: 1.59) and an average fetal hemoglobin (HbF) level of 10.59% (SD: 5.64). The frequency of VOCs varied, with 30% of patients reporting no VOC in the past year, while 57% experienced two to three VOCs. Patients with higher VOC frequency (≥4 per year) reported significantly lower scores across all QoL domains except stiffness (p < 0.05). Higher VOC severity was associated with poorer sleep quality and social functioning (p < 0.05). Pain severity was also a critical determinant, with more than 55% of patients rating their last pain episode as severe or extreme.

Conclusion

The study highlights the significant burden of pain and VOCs on the quality of life in adult SCD patients. Frequent VOCs and chronic complications such as avascular necrosis and gallstones were major contributors to reduced HRQoL. Findings emphasize the need for comprehensive, multidisciplinary care approaches targeting pain management, psychological support, and functional independence. The ASCQ-Me tool proved valuable for identifying specific domains of impairment and guiding patient-centered interventions. Future research should focus on longitudinal studies to explore the efficacy of tailored interventions and address the ongoing challenges faced by this vulnerable population.

## Introduction

Sickle cell disease (SCD) is a genetic blood disorder marked by the production of abnormal hemoglobin S, which leads to red blood cell deformities, chronic hemolysis, and frequent vaso-occlusive crises (VOC) [1, 2]. The disease predominantly affects populations in Sub-Saharan Africa, the Middle East, and India, and it remains a major public health concern due to its associated complications and reduced life expectancy [3, 4]. Despite medical advancements improving survival rates, adults with SCD continue to face substantial challenges, particularly in terms of quality of life (QoL) [4].

In patients with SCD, the QoL is often compromised due to recurrent VOCs, chronic pain, and multiple organ complications [5]. Pain is a predominant and recurring issue in SCD, manifesting both as acute pain episodes due to VOC and as chronic pain stemming from musculoskeletal or organ damage [6]. These pain episodes lead to significant physical and psychological stress, disrupting everyday life, social relationships, and emotional well-being [7]. Additionally, chronic complications such as gallstones, avascular necrosis, and cerebrovascular events can further exacerbate pain and functional limitations, adversely impacting the health-related quality of life (HRQoL) [8,9].

To quantify and assess the quality of life in SCD patients, standardized tools such as the Adult Sickle Cell Quality of Life Measurement Information System (ASCQ-Me) have been utilized. The ASCQ-Me measures key domains such as pain impact, emotional well-being, sleep, and social functioning using a standardized scoring system [10,11]. This approach provides valuable insights into the specific domains affected by SCD, allowing for tailored interventions aimed at improving patient outcomes. However, despite these advances, there remain significant gaps in understanding the relationship between the frequency and severity of VOCs and the overall quality of life in adults with SCD [12].

This study seeks to investigate the quality of life in adult patients with SCD, focusing on key demographic and clinical variables influencing health outcomes. By employing the ASCQ-Me short form, the study aims to highlight critical areas where targeted interventions can enhance the HRQoL in this vulnerable population.

## Materials and methods

Study design and setting

This cross-sectional study was conducted at the Prince Sultan Oncology Center, King Salman Northwest Armed Forces Hospital, Tabuk, Saudi Arabia. The study aimed to assess the QoL in adult patients with SCD who were regularly followed in outpatient clinics between December 2019 and May 2020. Ethical approval for the study was obtained from the ethical committee of King Salman Armed Forces Hospital (R&REC2017-158), and informed consent was obtained from all participants prior to their inclusion.

Study population and data collection

The ASCQ-Me Short Form, a validated instrument, was used to assess the QoL of SCD patients. The ASCQ-Me questionnaire consists of five key domains: emotional impact, social functioning, pain impact, sleep impact, and joint stiffness impact. Each domain was assessed using a five-item questionnaire, and the pain impact domain was further classified into frequency, timing, and severity of SCD-related pain episodes. Additionally, the ASCQ-Me Medical History Checklist, comprising nine items related to treatments and complications of SCD, was used to evaluate participants’ health status comprehensively.

Data collection was carried out through structured one-on-one interviews, which were conducted by a trained health educator. The sociodemographic profile of each participant, including age, gender, marital status, education level, and employment status, was also collected during the interviews. All data collection procedures followed the standard protocols outlined in the ASCQ-Me manual to ensure consistency and accuracy.

Scoring and standardization

The responses from the ASCQ-Me questionnaires were scored according to the standardized guidelines provided by the developers of the instrument. The domain scores were transformed into T-scores, standardized to a mean of 50 with a standard deviation of ±10, facilitating a consistent comparison across domains. Data compilation and scoring followed the strict guidelines outlined in the ASCQ-Me user manual to maintain data integrity.

Statistical analysis

Data were entered and analyzed using IBM SPSS Statistics software, version 26 (IBM Corp., Armonk, NY). Descriptive statistics, including frequencies, percentages, mean, and standard deviation, were utilized to summarize the sociodemographic characteristics and key variables. The results were presented in both tabulated and graphical formats for clarity.

For inferential analysis, the SCD patients were stratified based on the number of VOCs experienced in the past 12 months. Patients were classified into two groups: those with 0 to three VOCs and those with ≥4 VOCs. The Mann-Whitney U test was employed to compare the ASCQ-Me domain scores between these two groups. Additionally, VOC severity was stratified using a benchmark T-score of 50 with a standard deviation of 10. Patients with pain episode severity scores ≥55 were classified as having more severe VOCs, while those with scores <55 were classified as having less severe VOCs. The Mann-Whitney U test was again utilized to determine differences between domain scores according to VOC severity. A p-value of ≤0.05 was considered statistically significant for all analyses.

## Results

Demographics

A total of 53 adult patients diagnosed with SCD were surveyed using the ASCQ-Me Short Form questionnaire. The participants were divided into five age groups, with the highest representation from the 25-34 year age group (50%). Gender representation was nearly equal, with 50.9% males and 49.1% females. Notably, 69% of respondents were single, and most were students or unemployed, leading to a prevalence in the lower income categories. Over 65% of participants had attained a secondary level or higher education. Additionally, 76% reported a family history of the disease. This demographic profile is detailed in Table 1.

**Table 1:** Demographic and socioeconomic characteristics of the study participants The table displays the distribution of adult SCA participants by age, gender, marital status, employment, residence, income, education, and family history. Note: total patients = 53. Some questions had missing responses, resulting in variations in the number of responses per question.

Characteristics		Frequency	Percentage
Age	Less than 18 years	7	13%
	18-24 years	10	18.5%
	25-34 years	27	50%
	35-44 years	9	16.7%
	≥ 45 years	1	1.9%
Gender	Male	27	50.9%
	Female	26	49.1%
Marital status	Single	29	69%
	Married	10	23.8%
	Divorced	3	7.1%
Employment status	Military	6	14%
	Non-military	4	9.3%
	Student	19	44.2%
	Unemployed	14	32.6%
Residence status	Owner	24	58.5%
	Renter	17	41.5%
Monthly income (in Saudi Riyal (SAR))	Less Than 2K SAR	13	31%
	2K-4K SAR	6	14.3%
	4K-6K SAR	6	14.3%
	6K-8K SAR	1	2.4%
	8K-10K SAR	8	19%
	10K-12K SAR	7	16.7%
	More than 14K SAR	1	2.4%
Education	Illiterate	1	2.2%
	Primary level	5	10.9%
	Intermediate level	10	21.7%
	Secondary level	14	30.4%
	College level	16	34.8%
Family history	Yes	29	76.3%
	No	9	23.7%

Frequency of complications

The study assessed the frequency of common SCD-related complications. Chronic pain was the most frequently reported complication, affecting 31% of the respondents, followed by gallstones (26%) and avascular necrosis (9%). Complications like stroke and pulmonary hypertension had lower prevalence rates, with only 2% experiencing thromboembolic disease or priapism. The detailed data are provided in Table 2.

**Table 2:** Frequency of complications in adult sickle cell disease (SCD) patients The table lists the common complications experienced by adult SCD participants, including chronic pain, gallstones, avascular necrosis, and stroke. The table presents the frequency and percentage of each complication, highlighting the prevalence of specific health issues within this population.

Complications	Frequency	Percentage
Chronic pain	17	31%
Gallstones	14	26%
Avascular necrosis	5	9%
Stroke (history of stroke)	3	6%
Pulmonary hypertension	1	2%
Thrombo-embolic disease (AH)	1	2%
Priapism	1	2%

Laboratory findings

Several hematological parameters were measured, revealing an average hemoglobin level of 8.63 g/dL (SD: 1.59) and a hematocrit of 25.06% (SD: 4.66). Fetal hemoglobin (HbF) levels averaged 10.59% (SD: 5.64). Additionally, the study recorded bilirubin levels, with indirect bilirubin at a mean of 33.02 µmol/L (SD: 20.26). Reticulocyte counts averaged 11.96% (SD: 5.28). Full laboratory results are summarized in Table 3.

**Table 3:** Laboratory findings of adult sickle cell disease (SCD) patients The table summarizes key laboratory findings for adult SCD participants, including Hb, HCT, HbF, total bilirubin, indirect bilirubin, and reticulocyte count. The table presents the mean values and standard deviations (SD), providing insight into the typical hematologic and biochemical profile of the study population.

Laboratory findings	Mean	S.D.
Hemoglobin (Hb)	8.6296	1.59358
Hematocrit (HCT)	25.0556	4.65576
Fetal hemoglobin (HbF)	10.5926	5.64189
Total bilirubin	44.8519	26.82537
Indirect bilirubin	33.0185	20.25823
Retics count	11.963	5.28423

The ASCQ-Me SCD Medical History Checklist

The study incorporated the ASCQ-Me SCD Medical History Checklist to assess the respondents’ health status. The average checklist score was 1.2 ± 0.18, indicating a generally favorable health status among the participants. More than 50% of the sample reported no health history indicators, while 10% reported three or more indicators, reflecting poorer health outcomes. The most common indicators included hip or shoulder damage (31%), daily use of pain medication (31%), and regular blood transfusions (22%). The detailed breakdown is presented in Table 4.

**Table 4:** ASCQ-Me SCD medical history checklist The table displays the responses of adult SCA participants to nine questions from the ASCQ-Me SCD Medical History Checklist, covering treatments and conditions associated with SCD. It provides insight into the health status of participants by documenting their history of complications and treatments related to SCD. ASCQ-Me: Adult Sickle Cell Quality of Life Measurement Information System; SCD: sickle cell disease

Questions	Frequency	Percentage
Has a doctor or nurse ever told you that you have damage to your hip or shoulder due to sickle cell disease?	17	31%
Do you take pain medicine every day for your sickle cell disease?	17	31%
Do you get regular blood transfusions for your sickle cell disease?	12	22%
Has your spleen either been removed or seriously damaged due to sickle cell disease?	7	13%
Has a doctor or nurse ever told you that you have lung damage?	6	11%
Have you ever had open sores on your legs or feet (leg ulcers)?	2	4%
Has a doctor or nurse ever told you that you have eye damage called retinopathy?	2	4%
Has a doctor or nurse ever told you that you have kidney damage?	1	2%
Has a doctor or nurse ever told you that you have had a stroke?	1	2%

Pain episode data

The frequency and timing of pain episodes revealed that 30% of respondents had not experienced a pain episode in the past 12 months. However, more than half of the patients (57%) experienced two to three episodes. When asked about the timing of their last pain episode, the majority (40.7%) reported an episode within the last one to six months, and 33% had experienced an episode in the previous month. These findings are depicted in Table 5.

**Table 5:** Frequency and timing of pain attacks in adult sickle cell disease (SCD) patients The table presents the frequency of pain attacks (crises) over the past 12 months and the timing of the last pain episode among adult SCD participants. This table provides insights into the recurrence and recentness of pain crises, offering a perspective on pain burden and its impact on quality of life.

Questions	Frequency	Percentage
When was your last pain attack (crisis)?		
I did not have a pain attack (crisis) in the past 12 months	5	9.3%
0 times	11	20.4%
1 time	7	13%
2 times	14	25.9%
3 times	17	31.5%
When was your last pain attack (crisis)?		
I never had a pain episode	2	3.7%
More than 5 years ago	3	5.6%
1–5 years ago	3	5.6%
7–12 months ago	5	9.3%
1–6 months ago	22	40.7%
1–4 weeks ago	12	22.2%
Less than a week ago	6	11.1%
I have one right now	1	1.9%

Impact of pain on daily life

The impact of pain on daily life varied among respondents. Notably, 30% experienced significant interference, requiring help from family, friends, or healthcare workers, while 53% reported minimal or no interference. The average duration of the last pain episode was four to seven days (27.8%), with over 44% experiencing episodes lasting more than a week. The severity rating on a scale from 0 to 10 yielded an average score of 7.26 ± 2.36, indicating substantial pain intensity. This is detailed in Table 6.

**Table 6:** Impact and duration of the last pain attack in adult sickle cell disease patients The table illustrates the degree of interference caused by the most recent pain attack (crisis) on participants' daily activities and its duration. The table categorizes responses based on the level of support required and the length of the episode, including a mean severity rating on a scale of 0 to 10. This information highlights the intensity and prolonged nature of pain episodes and their impact on independence and quality of life.

Questions	Frequency	%age
How much did your last pain attack (crisis) interfere with your life?		
I’ve never had a pain attack (crisis)	3	5.6%
Not at all; I did everything I usually do	11	20.4%
I had to cut down on some things I usually do	15	27.8%
I could not do most things I usually do	9	16.7%
I could not take care of myself and needed some help from family or friends	5	9.3%
I could not take care of myself and needed constant care from family, friends, doctors or nurse.	11	20.4%
About how long did your most recent pain attack (crisis) last?		
I’ve never had a pain attack (crisis)	2	3.7%
Less than 1 hour	1	1.9%
1–12 hours	4	7.4%
13–24 hours	3	5.6%
1–3 days	5	9.3%
4–7 days	15	27.8%
1–2 weeks	14	25.9%
More than 2 weeks	10	18.5%
Patient rating of severity in last pain attack (0-10)	Mean ± SD	
	7.26 ± 2.36	

Composite scores of ASCQ-Me domains

The ASCQ-Me domains were analyzed and transformed into standardized T-scores. The scores indicated favorable outcomes in domains like emotional impact, sleep, social functioning, and stiffness, with all scoring above the benchmark average of 50. Pain impact scores aligned with the average benchmark, while pain episode severity was notably higher than average. The domain scores are summarized in Table 7.

**Table 7:** ASCQ-Me quality of life scores across domains The table displays mean, standard deviation (SD), median, and interquartile range (IQR) for various ASCQ-Me domains, including emotional impact, pain impact, sleep quality, social functioning, stiffness, pain episode frequency, and pain episode severity. This table provides a comprehensive overview of how different quality of life aspects are affected in adult SCA participants, with scores reflecting both central tendencies and variability.

ASCQ-Me	Mean	SD	Median	IQR
Emotional impact	53.9679	9.4	53.3	35.1-71.5
Pain impact	50.1925	11.6	49.9	30.5-69.3
Sleep	52.3585	7.8	51.1	41.1-61.1
Social functioning	53.1302	9.7	54	41.4-66.6
Stiffness	52.7547	10.6	51	32.3-69.7
Pain episode frequency	45.0189	12.2	47	32-62
Pain episode severity	56.6604	14.7	56	44.5-67.5

Vaso-occlusive crises frequency and ASCQ-Me domains

The frequency of VOCs in the past 12 months was categorized into two groups: those experiencing 0 to three VOCs and those with four or more. Patients with more frequent VOCs reported significantly poorer health-related quality of life across all domains except stiffness. The SCD medical history checklist also revealed that patients with frequent VOCs endorsed a higher number of health indicators. These associations are presented in Table 8.

**Table 8:** Comparison of ASCQ-Me scores by frequency of VOCs in the past year This table compares the quality of life scores across ASCQ-Me domains (emotional impact, pain impact, sleep, social functioning, stiffness) between adult sickle cell disease participants who experienced 0 to three vaso-occlusive crises (VOCs) and those with four or more VOCs in the past 12 months. The table includes mean, standard deviation (SD), median, Z-scores, and p-values, highlighting statistically significant differences in quality of life between the two groups.

ASCQ-Me domains	0-3 VOCs in the past 12 months				4 or more VOCs in the past 12 months				Z	p-value
	N	Mean	SD	Median	N	Mean	SD	Median		
Emotional impact	37	57.5	7.8	57.3	17	46.6	8.2	47.4	-3.86	0.000
Pain impact	37	54.1	9.8	54.9	17	42.0	11.0	44.4	-3.67	0.000
Sleep	37	54.8	7.3	55.3	17	47.2	6.4	49.7	-3.26	0.001
Social functioning	37	56.3	9.2	55.8	17	46.5	7.4	47.2	-3.65	0.000
Stiffness	36	53.9	10.1	51.85	17	50.4	11.5	48.1	-1.42	0.156
SCD medical history checklist	37	0.8	0.9	1	17	2.1	1.6	2	-2.73	0.006

Vaso-occlusive crises severity and ASCQ-Me domains

The severity of VOCs was classified based on a T-score benchmark of 50. Higher VOC severity was associated with poorer quality of life in all ASCQ-Me domains, with statistically significant differences observed in sleep and social functioning (p < 0.05). The medical history checklist showed that patients with more severe VOCs endorsed a greater number of indicators. The findings are detailed in Table 9.

**Table 9:** Comparison of ASCQ-Me scores by severity of VOCs in adult SCA patients The table compares quality of life scores in ASCQ-Me domains (emotional impact, pain impact, sleep, social functioning, stiffness) between adult SCA participants experiencing less severe versus more severe vaso-occlusive crises (VOCs). Includes mean, standard deviation (SD), median, Z-scores, and p-values to identify significant differences. This table provides insights into how VOC severity influences various quality of life aspects, with statistically significant findings in sleep, social functioning, and the SCD medical history checklist.

ASCQ-Me domains	Less severe VOCs				More severe VOCs				Z	p-value
	N	Mean	SD	Median	N	Mean	SD	Median		
Emotional impact	16	56.0	8.1	54.4	37	53.1	9.8	53.3	-0.97	0.334
Pain impact	16	54.1	6.7	51.2	37	48.5	12.8	45.7	-1.93	0.054
Sleep	16	57.0	6.5	56.7	37	50.4	7.6	51.1	-2.85	0.004
Social functioning	16	56.6	7.1	55.8	37	51.6	10.4	50.5	-2.24	0.025
Stiffness	16	55.0	7.7	51	37	51.8	11.6	51.0	-1.02	0.309
SCD medical history checklist	16	0.8	1.3	0	37	1.4	1.3	1.0	-2.08	0.038

## Discussion

Burden of pain and its multifaceted impact on quality of life

Pain stands as the most significant and persistent complication in adults with SCD, affecting nearly all aspects of daily life. The current study’s findings are consistent with a substantial body of literature indicating that pain in SCD often transitions from episodic to chronic, impacting up to 40% of adult patients. Furthermore, SCD-related pain is associated with considerable morbidity and an elevated risk of mortality. While pain episodes in SCD are generally acute, they tend to increase in frequency with age, and in adults, pain frequently becomes a daily occurrence, closely resembling a chronic pain syndrome. [6,12]. Chronic pain syndromes in SCD often develop as a result of avascular necrosis, bone infarction, and neuropathy, further diminishing physical and social functioning [13]. Neuropathic pain, a significant subtype of pain in SCD, has been reported by Orhurhu et al. to occur at notable levels and is closely associated with poor emotional health and reduced HRQoL [6]. Additionally, repeated acute pain episodes contribute to an ongoing cycle of pain and psychological distress, leading to anxiety, depression, and decreased self-efficacy [14,15]. The cumulative effect of these pain episodes has been noted to impact general health, vitality, and bodily pain domains, further amplifying the burden of pain on quality of life [12].

The study also revealed substantial interference with daily activities, which is consistent with the findings of McClish et al., who noted that up to 60% of adults with SCD reported limitations in their ability to perform routine tasks due to pain [16]. Moreover, Glassberg et al. highlighted that the frequency of pain episodes is a key determinant of emergency department utilization, further illustrating the burden of inadequate pain management [17]. These findings emphasize the need for a more comprehensive and individualized approach to pain management in SCD, integrating pharmacological, psychological, and social interventions. Additional findings support the impact of pain on both social and emotional domains, which are vital to the overall well-being of SCD patients [12].

Frequency and severity of VOCs: the tipping point in QoL deterioration

Our study demonstrated a significant decline in all QoL domains in patients with higher frequencies of VOCs, particularly in pain impact, social functioning, and sleep. This is supported by previous studies, such as those conducted by Powars et al., which have shown that VOC frequency is not only a predictor of mortality but also a key indicator of reduced HRQoL [18]. Vaso-occlusive crises often lead to acute hospital admissions, increased opioid use, and longer recovery periods, further disrupting social and occupational functioning [19]. Findings from recent literature further affirm that VOC frequency is associated with missed work and school days, impacting both the personal and professional lives of individuals [12].

Furthermore, the impact of frequent VOCs extends beyond immediate pain. Studies by Ballas and Lusardi indicate that patients with recurrent VOCs exhibit greater cumulative organ damage, increased risk of renal dysfunction, and a greater incidence of complications like pulmonary hypertension [20]. These complications compound the negative effects of pain, creating a vicious cycle of physical impairment and psychological distress. Similar observations have been reported with impacts on work productivity, where frequent VOCs correlate with higher absenteeism and impaired work performance [12].

Psychosocial and economic challenges

The psychosocial burden of SCD cannot be underestimated. In the present study, more than half of the participants were unemployed or students and 69% were unmarried, reflecting the pervasive socioeconomic challenges faced by SCD patients. Similar findings were reported by Williams et al., where unemployment and economic instability were prevalent among adults with SCD, largely due to the frequent need for medical care and absenteeism from work or school [19]. The lack of stable income further limits access to adequate healthcare, pain management, and social support, exacerbating the disease’s impact. Data from previous studies indicate that these financial constraints are linked with poorer outcomes, including higher hospital readmission rates and greater healthcare resource utilization [12].

Psychologically, the chronic nature of SCD predisposes individuals to depression, anxiety, and diminished coping abilities. Studies by Levenson et al. highlight that the prevalence of depression and anxiety in SCD patients can reach up to 27.6% and 6.5%, respectively, with higher rates of depressive symptoms linked to lower levels of social support and higher pain severity [21, 22]. Addressing these mental health challenges is critical, as untreated psychological distress is associated with increased pain perception and healthcare utilization. Additionally, mental health challenges impact interpersonal relationships and education, with nearly half of patients reporting strained personal connections and altered educational trajectories due to SCD [12].

Impact of chronic complications on long-term outcomes

Chronic complications such as avascular necrosis, retinopathy, and gallstones play a crucial role in shaping the long-term health outcomes of SCD patients. Avascular necrosis, reported by 9% of our study participants, is a well-documented cause of chronic pain and disability in adults with SCD [23]. Data from previous studies indicate a high prevalence of progression from asymptomatic osteonecrosis to symptomatic disease and femoral head collapse. Mont et al. demonstrated that nearly 50% of adult SCD patients develop avascular necrosis, with the majority experiencing progressive joint deterioration, often leading to surgical interventions and prolonged rehabilitation [24]. These findings highlight the importance of early diagnosis and preventive measures to mitigate joint damage.

Pulmonary complications, including pulmonary hypertension, remain a significant concern in SCD management. Pulmonary hypertension, which was observed in 2% of the participants in this study, is associated with a marked increase in morbidity and mortality. In a large-scale cohort study by Castro et al., pulmonary hypertension was identified as a major predictor of early mortality in adult SCD patients, underscoring the need for routine screening and early intervention [25].

Sleep disturbances and cognitive implications

Sleep disturbances are another critical issue in SCD, with significant implications for both physical and cognitive health. This study reported poor sleep quality in participants with frequent VOCs, which aligns with prior research showing that up to 60% of adults with SCD suffer from sleep-related issues [26]. Pain-induced sleep disruption is known to contribute to fatigue, impaired concentration, and diminished executive functioning. Studies by Rhodes et al. have further explored the association between poor sleep and cognitive decline in SCD patients, indicating that chronic pain and sleep disturbances contribute to the accelerated cognitive aging observed in this population [27]. Recent literature also emphasizes that disrupted sleep cycles significantly correlate with the frequency and intensity of VOCs, impacting mental health outcomes [12].

The role of comprehensive care and patient-centered interventions

The findings of this study highlight the urgent need for a comprehensive, multidisciplinary approach engaging hematologists, pain specialists, pharmacists, and social workers to enhance the quality of life outcomes for patients with SCD. Patient-centered interventions targeting pain management, emotional well-being, and functional independence are paramount. The ASCQ-Me instrument has proven to be a valuable tool for identifying key domains of impairment and monitoring patient progress over time [10]. Evidence-based pain management strategies should incorporate both pharmacologic (e.g., individualized opioid regimens) and non-pharmacologic approaches (e.g., cognitive behavioral therapy, mindfulness) to address the multifaceted nature of pain in SCD [28, 29].

Furthermore, social and psychological support systems must be integrated into care models to help patients cope with the emotional and social challenges posed by SCD. Programs focusing on vocational rehabilitation, patient education, and peer support have demonstrated positive effects on employment outcomes and self-efficacy in this patient population [7, 30]. Health policy efforts should also aim to improve access to comprehensive care, reduce disparities in healthcare access, and promote equity in pain management services.

Limitations and future research

While this study provides valuable insights into the QoL challenges faced by adults with SCD, several limitations must be acknowledged. The relatively small sample size and cross-sectional design limit the ability to draw causal inferences. Additionally, the self-reported nature of the ASCQ-Me responses may introduce bias. Future research should aim to include larger and more diverse patient cohorts, utilize longitudinal designs to examine temporal relationships between VOCs and QoL, and explore the efficacy of tailored interventions.

## Conclusions

This study highlights the significant impact of frequent and severe VOCs, chronic pain, and complications on the quality of life in adult patients with SCD. The findings emphasize the need for comprehensive, multidisciplinary care strategies that address pain management, psychological support, and functional independence. The ASCQ-Me tool proved effective in identifying key domains of impairment, enabling targeted interventions. Moving forward, integrated care models focusing on both physical and psychosocial aspects are essential to improving the health outcomes and overall well-being of SCD patients.
